# An inflammatory biomarker panel for prediabetes classification using interpretable machine learning

**DOI:** 10.1371/journal.pone.0341195

**Published:** 2026-03-16

**Authors:** Maher Maalouf, Maram Tammam, Sana Kurungadan, Asmaa Alsereidi, Muhammad Afzal, Herbert F. Jelinek

**Affiliations:** 1 Department of Management Science and Engineering, Khalifa University, Abu Dhabi, United Arab Emirates; 2 Department of Mathematics and Computer Science, Khalifa University, Abu Dhabi, United Arab Emirates; 3 Department of Medical Sciences, Khalifa University, Abu Dhabi, United Arab Emirates; 4 Health Engineering Innovation Group, Khalifa University, Abu Dhabi, United Arab Emirates; University of Montenegro-Faculty of Medicine, MONTENEGRO

## Abstract

**Objective:**

Prediabetes is a silent condition that often goes undetected. However, timely interventions could prevent its progression to type 2 diabetes. Traditional glycemic markers, such as hemoglobin A1c (HbA1c), have limitations, creating a need for new diagnostic biomarkers. In this study, our objective was to develop an interpretable machine learning model using biomarkers related to oxidative stress, inflammation, and lipid metabolism to classify prediabetes independently of traditional glycemic markers, such as HbA1c. We also compared multiple biomarker panels to determine which biomarkers offer the highest predictive accuracy.

**Methods:**

We developed and validated interpretable machine learning models using clinical and biomarker data from 545 participants (405 healthy controls and 140 with prediabetes). To ensure robust and generalizable findings, we employed a nested cross-validation technique, managed feature collinearity using the variance inflation factor (VIF), and interpreted the final model with Shapley Additive exPlanations (SHAP) [Kapoor S, Narayanan A. Patterns. 4(9):100804 (2023); Vabalas A, et al. PLoS One. 14(11):e0224365 (2019); Lundberg SM, Lee SI. Adv Neural Inf Process Syst. 30:4768–77 (2017)].

**Results:**

Our approach identified a distinct panel of inflammatory biomarkers (IL-10, IGF-1, and CRP) capable of classifying prediabetes independently of traditional glycemic markers. This non-glycemic model achieved a promising Area Under the Curve (AUC) of 0.711 on holdout validation, establishing inflammation as a key and measurable indicator of early metabolic dysfunction.

**Conclusion:**

Our findings introduce a novel panel of inflammatory biomarkers that show promise in the identification of prediabetes independently of traditional glucose-based measures. By highlighting inflammation as an early indicator of metabolic dysfunction, this approach may enhance precision in the detection of prediabetes. Longitudinal studies with larger and more diverse populations are essential to clinically validate these biomarkers and confirm their value in improving the early diagnosis and management of metabolic health.

## 1 Introduction

Prediabetes is a common metabolic disorder characterized by elevated blood glucose levels below the threshold for the diagnosis of type 2 diabetes mellitus (T2DM). Almost 590 million adults worldwide live with diabetes and more than 630 million people are estimated to have prediabetes. This underscores a major public health crisis and a critical window for early intervention and prevention [1–3]. Despite its health consequences, including the increased risks of cardiovascular disease, kidney disease, and neuropathy, prediabetes is often undiagnosed because it usually occurs without overt symptoms [4–8].

Traditionally, the diagnosis of prediabetes is based mainly on fasting plasma glucose (FPG) and glycated hemoglobin (HbA1c) tests. However, these methods may not reliably detect early metabolic changes and can sometimes misclassify individuals [9–11]. Therefore, researchers are increasingly exploring alternative diagnostic approaches, such as the evaluation of cardiovascular risk factors and patient subphenotypes, to improve predictive accuracy and early risk identification [12,13].

Recent research has highlighted the importance of biomarkers associated with early metabolic disturbances, independent of traditional glucose markers. Systematic reviews highlight ongoing efforts to identify biomarkers that could improve our understanding and early detection of type 2 diabetes [14]. Researchers now recognize biological processes, such as chronic inflammation, mitochondrial dysfunction, and oxidative stress, as crucial early disruptions that precede overt glycemic disorders [15–19]. Biomarkers related to these processes, mainly markers of oxidative DNA damage, have the potential for early detection and intervention in metabolic disorders [20].

Machine learning (ML), especially interpretable algorithms, provides a powerful approach to identifying complex interactions among biomarkers related to prediabetes [21]. However, current studies based on ML often rely heavily on traditional glucose-based biomarkers, restricting the ability to uncover novel metabolic markers [22–24].

Recent studies in predictive modeling have emphasized the predictive potential of biomarkers. For example, these biomarkers have been applied to directly predict the risk of diabetes using machine learning [25]. In addition, these techniques have been shown to be more comprehensive in their ability to evaluate disease progression using inflammatory and related markers in other chronic conditions that frequently coexist with metabolic disorders, such as depression [26] and cardiac autonomic neuropathy [27].

To address this gap, our study uses a targeted ML approach specifically designed to evaluate the predictive value of non-glycemic biomarkers. We systematically examine biomarkers associated with mitochondrial function, inflammation, and oxidative stress, which have previously been shown to be relevant for the prediction of chronic disease [28–30]. By applying robust statistical methods and interpretable machine learning approaches (SHAP), our objective is to identify biomarkers that predict prediabetes independently of HbA1c. Clarifying the independent predictive value of these biomarkers could significantly improve early risk identification and enable more targeted preventive interventions in clinical practice [31].

## 2 Methods

### 2.1 Study design and data source

This study is a secondary analysis of data from the DiabHealth rural diabetes screening clinic, which prospectively collected participant data between 2002 and 2015. The original data collection received full ethical approval from the Charles Sturt University Human Research Ethics Committee (CSU HREC; protocol 2006/042), and all participants provided their written informed consent [32]. For the present research, the pre-existing, de-identified dataset was accessed in January 2025 solely for statistical analysis; no participant contact, recruitment, or data collection occurred after 2015. Data were cleaned for the parent study using established protocols described by Jelinek et al. [33], which gives a complete analytic dataset for the variables examined here.

### 2.2 Study population and outcome definition

The analytical cohort for this study was derived from the parent DiabHealth dataset, which initially included 847 participants. From this cohort, individuals with a prior diagnosis of diabetes were first excluded, resulting in a sample of 604 individuals. To further refine the cohort to specifically isolate the prediabetic state and minimize the confounding effects associated with advanced age, we applied additional exclusion criteria. Participants with fasting glucose levels ≥ 7.0 mmol/L (indicative of previously undiagnosed diabetes) or those over 85 years of age were removed. This final selection process yielded a study cohort of 545 participants. Prediabetes was defined according to the criteria of the American Diabetes Association (ADA) [5]: participants with fasting screening glucose between 5.6 and 6.9 mmol/L (inclusive) were classified as ’prediabetes’ (n = 140), and those with levels less than 5.6 mmol/L were classified as ’control’ (n = 405). Baseline characteristics were compared between groups using Welch’s t-test for continuous variables and the $\chi^{2}$-test for categorical variables, implemented using Python’s SciPy library.

### 2.3 Biomarker panels for analysis

The biomarkers in this study were selected based on their established involvement in metabolic disorders and their potential predictive value for T2DM (Table 1). The biomarker panel included biomarkers of lipid metabolism (triglycerides, total cholesterol [TC], high-density lipoprotein [HDL], low-density lipoprotein [LDL]), oxidative stress (GSH, GSSG, GSH/GSSG, 8-OHdG), mitochondrial function (Humanin, MOTS-c, p66Shc), inflammation (CRP, IL-6, IL-1*β*, IL-10, MCP-1, IGF-1), the glycemic marker HbA1c, and the demographic variable age.

**Table 1:** Description of biomarkers and clinical features used in the study.

Category	Variable	Description
**Lipid Panel (blood)**	Triglyceride	Fat in the blood; high levels increase the risk of heart disease and diabetes.
	TC	Total cholesterol; higher levels can increase heart disease risk.
	HDL	High-density lipoprotein (“good cholesterol”); helps protect against heart disease by removing harmful cholesterol.
	LDL	Low-density lipoprotein (“bad cholesterol”); higher levels increase heart disease risk.
**Oxidative Stress (blood or urine)**	GSH	Reduced glutathione (GSH); an antioxidant that protects cells from damage.
	GSSG	Oxidized glutathione (GSSG); higher levels indicate increased cell stress.
	GSH/GSSG	Ratio indicating antioxidant balance; lower ratios mean higher oxidative stress.
	8-OHdG	Marker of oxidative DNA damage, indicating stress on cells.
**Mitochondrial Function (blood)**	Humanin	Protective mitochondrial protein supporting cell health and insulin sensitivity.
	MOTS-c	Mitochondrial protein regulating metabolism and insulin sensitivity.
	p66Shc	Protein linked to aging; involved in the cell’s response to stress.
**Inflammatory (blood)**	CRP	C-reactive protein; a general indicator of inflammation.
	IL-6	Interleukin-6; linked to inflammation, higher in metabolic disorders.
	IL-1*β*	Interleukin-1 beta; promotes inflammation during immune responses.
	IL-10	Interleukin-10; reduces inflammation and controls immune responses.
	MCP-1	Monocyte chemoattractant protein-1; attracts immune cells during inflammation.
	IGF-1	Insulin-like growth factor-1; regulates insulin sensitivity and inflammation.
**Glycemic Marker (blood)**	HbA1c	Glycated hemoglobin; shows average blood sugar over the past 2–3 months.
**Demographics**	Age	Participant’s age in years.

Inflammatory biomarkers such as interleukin-6 (IL-6), C-reactive protein (CRP), and interleukin-1*β* (IL-1*β*) were included because they link chronic low-grade inflammation to insulin resistance through activation of the c-Jun N-terminal kinase (JNK) and I*κ*B kinase *β* (IKK*β*)–nuclear factor *κ*B (NF-*κ*B) signaling pathways [15,17]. Oxidative stress biomarkers, particularly 8-hydroxy-2’-deoxyguanosine (8-OHdG) and the reduced-to-oxidized glutathione ratio (GSH/GSSG), were selected based on evidence that systemic oxidative damage precedes the clinical onset of hyperglycemia [18,20]. Mitochondrial-derived peptides, including Humanin and the mitochondrial open reading frame of 12S rRNA type-c (MOTS-c), were included as emerging metabolic regulators that modulate systemic insulin sensitivity [29].

To systematically assess predictive capacity and isolate non-glycemic signals, variables were grouped into seven biomarker panels (Table 2). Three panels captured single biological domains: (i) cholesterol_only (lipid biomarkers: triglycerides, TC, HDL, LDL), (ii) oxidative_only (oxidative stress biomarkers: GSH, GSSG, GSH/GSSG, 8-OHdG), and (iii) inflammatory_only (inflammatory biomarkers: CRP, IL-6, IL-1*β*, IL-10, MCP-1, IGF-1). Two additional panels evaluated conventional risk markers in isolation: (iv) HbA1c_only (glycated hemoglobin) and (v) age_only.

**Table 2:** Biomarker panels used in analysis.

Panel Name	Biomarker Combination
cholesterol_only	Cholesterol biomarkers only
oxidative_only	Oxidative stress biomarkers only
inflammatory_only	Inflammatory biomarkers only
HbA1c_only	Glycemic marker (HbA1c) only
age_only	Age only
all_biomarkers	All biomarkers excluding HbA1c and age
all_features	All biomarkers combined with age

The remaining panels were defined to contrast non-glycemic biomarker information with more traditional predictors. The all_biomarkers panel combines all non-glycemic biomarkers (lipid, oxidative stress, mitochondrial function, and inflammatory markers), explicitly excluding HbA1c and age, reflecting the predictive capacity of biological biomarkers alone. The all_features panel includes all biomarkers (non-glycemic biomarkers and HbA1c) together with age. Clinical variables such as hypertension status, history of cardiovascular disease, and gender were deliberately excluded from all panels to focus on the predictive contribution of biomarkers and age. Future studies could integrate these clinical and demographic factors into extended risk prediction models.

### 2.4 Machine learning and statistical analysis

We developed a robust predictive model using Python with libraries including scikit-learn, pandas, statsmodels, XGBoost, LightGBM, and SHAP. The analysis strictly adhered to best practices to avoid data leakage and ensure external validity and generalizability of the findings [34,35]. The dataset was divided into a main set (80% of the data) for initial model development and a holdout set (20%) for final evaluation.

#### 2.4.1 Model evaluation and hyperparameter tuning.

To ensure robust model development and evaluation, we applied nested cross-validation consisting of two levels: an outer five-fold cross-validation for model evaluation and an inner three-fold cross-validation within each outer fold for hyperparameter tuning. Hyperparameters were optimized using grid search (GridSearchCV) by maximizing the Area Under the Receiver Operating Characteristic Curve (AUC). The final performance metrics are the aggregated predictions from each test fold, providing unbiased estimates of generalization performance.

#### 2.4.2 Data processing pipeline.

All preprocessing steps were encapsulated within a scikit-learn pipeline object and applied independently inside each training fold to prevent data leakage [35]. The pipeline included:

- **Multicollinearity reduction:** A custom transformer iteratively removed the feature with the highest variance inflation factor (VIF) until all remaining features had a VIF below 5.0, resulting in a simpler, more interpretable model free of highly correlated variables [36].
- **Standardization:** Numeric features were standardized (mean = 0, standard deviation = 1) using StandardScaler, and categorical features encoded with OneHotEncoder.

#### 2.4.3 Model training and selection.

We evaluated four machine learning algorithms: Logistic Regression for interpretability [37], Random Forest for robustness against noise and outliers [38], and LightGBM [39] and XGBoost [40] for computational efficiency and high predictive performance [41]. The pipeline adhered strictly to best practices to prevent data leakage and ensure the generalizability of the model [34,35]. To manage class imbalance, we configured Logistic Regression, Random Forest, and LightGBM to use class_weight = ‘balanced’, while for XGBoost we used an adaptive scale_pos_weight based on the class distribution in each training fold. The hyperparameter grids explored are detailed in Table 3. The final model and the biomarker panel were selected on the basis of the highest mean AUC obtained during cross-validation. The class imbalance between controls and prediabetic subjects was addressed by class weighting, without the application of matching or additional imbalance adjustment techniques.

**Table 3:** Hyperparameter grids used for optimization.

Algorithm	Hyperparameter grid
Logistic Regression	penalty: [‘l1’, ‘l2’]; C: [0.1, 1, 10]
Random Forest	n_estimators: [100, 200]; max_depth: [7,17]
XGBoost	learning_rate: [0.05, 0.1]; n_estimators: [100, 200]
LightGBM	learning_rate: [0.05, 0.1]; n_estimators: [100, 200]

#### 2.4.4 Final model calibration and interpretation.

The selected final model was re-trained in the entire training set and calibrated using isotonic regression (calibratedClassifierCV). Performance was evaluated on the independent holdout set. Feature importance was assessed using Shapley Additive exPlanations (SHAP), a method that quantifies the contribution of each feature to model predictions [42]. SHAP calculations were based on a background sample of 100 observations, using the general shap.Explainer to automatically select the appropriate explainer type.

## 3 Results

### 3.1 Baseline characteristics

The final analytical cohort consisted of 405 controls (74.3%) and 140 individuals with prediabetes (25.7%). The prediabetic group was significantly older (p = 0.002) and exhibited significant differences in markers of oxidative stress (GSSG, p = 0.002; GSH/GSSG ratio, p < 0.001) and mitochondrial function (MOTS-c, p = 0.002; p66Shc, p = 0.001) compared to controls. As expected, the glucose and HbA1c levels in the screen were significantly higher in the prediabetes group (p < 0.001 for both). Hypertension status, cardiovascular disease history, and gender did not differ significantly between groups (all p > 0.05) and therefore were not included as predictors in subsequent modeling, as Table 4 shows.

**Table 4:** Baseline characteristics of the study population (n = 545).

Variable (Unit)	Controls (n = 405)	Prediabetes (n = 140)	P-value
Age (years)	62.90±13.32	66.81±10.69	0.002
Gender (F/M)	248/157	75/65	0.112
CVD History (yes/no)	160/245	59/81	0.584
Hypertension Status (yes/no)	160/245	62/78	0.322
Screen Glucose (mmol/L)	4.86±0.45	6.06±0.38	<0.001
HbA1c (%)	5.74±0.36	5.87±0.42	<0.001
Triglyceride (mmol/L)	1.31±0.62	1.33±0.61	0.728
TC (mmol/L)	5.21±0.93	5.30±1.01	0.327
HDL (mmol/L)	1.49±0.41	1.49±0.40	0.965
LDL (mmol/L)	3.08±0.78	3.17±0.78	0.221
CRP (mg/L)	2.67±2.27	2.46±2.33	0.342
IL-6 (pg/mL)	21.45±13.05	21.55±17.90	0.942
IL-1*β* (pg/mL)	16.96±13.51	15.68±13.26	0.334
IL-10 (pg/mL)	88.72±110.43	76.50±97.79	0.246
MCP-1 (pg/mL)	216.80±77.48	215.96±63.85	0.908
IGF-1 (ng/mL)	340.23±264.41	442.90±473.50	0.002
8-OHdG (ng/mL)	169.88±98.17	184.00±115.60	0.162
GSH (*μ*M)	1738.55±472.98	1658.38±495.35	0.088
GSSG (*μ*M)	327.67±165.65	377.16±166.15	0.002
GSH/GSSG	7.04±5.22	5.01±2.47	<0.001
Humanin (ng/mL)	226.34±108.72	248.53±131.96	0.050
MOTS-c (ng/mL)	627.22±212.08	705.51±343.64	0.002
p66Shc	50.98±12.61	46.78±13.62	0.001

Data are mean ± SD or n/n. P-values were calculated using Welch’s t-test for continuous variables and the chi-square test for categorical variables (Gender, CVD History, Hypertension Status).

### 3.2 Model performance and selection

After applying a multicollinearity threshold (VIF < 5), our analysis identified a LightGBM model using the inflammatory_only biomarker panel as the optimal classifier. It achieved the highest mean area under the curve (AUC = 0.743) across cross-validation folds, outperforming other biomarker combinations (Table 5).

**Table 5:** Performance summary of the best model for each biomarker panel (VIF < 5).

Biomarker Panel	Best Model	Mean AUC	95% CI for AUC	Mean F1 Score
inflammatory_only	LightGBM	0.743	0.686–0.802	0.458
all_features	LightGBM	0.734	0.684–0.792	0.441
all_biomarkers	LightGBM	0.734	0.684–0.792	0.441
oxidative_only	RandomForest	0.717	0.679–0.749	0.405
cholesterol_only	RandomForest	0.630	0.555–0.704	0.369
HbA1c_only	LightGBM	0.599	0.543–0.649	0.390
age_only	LogisticRegression	0.589	0.552–0.622	0.410

Performance metrics from 5-fold cross-validation with VIF < 5. The best model for each panel was selected based on the highest Mean AUC. Metrics are rounded to three decimal places

The inflammatory_only (LightGBM) panel produced the highest mean AUC (0.743). Although its performance was not statistically distinguishable from the all_features, all_biomarkers and oxidative_only panels due to overlap of the 95% confidence intervals, all these panels were significantly better than the baseline models such as HbA1c_only and age_only, whose confidence intervals did not overlap with those four top panels. Therefore, the inflammatory_only panel was selected as the final model due to its high predictive accuracy and fewer variables.

**Fig 1:**
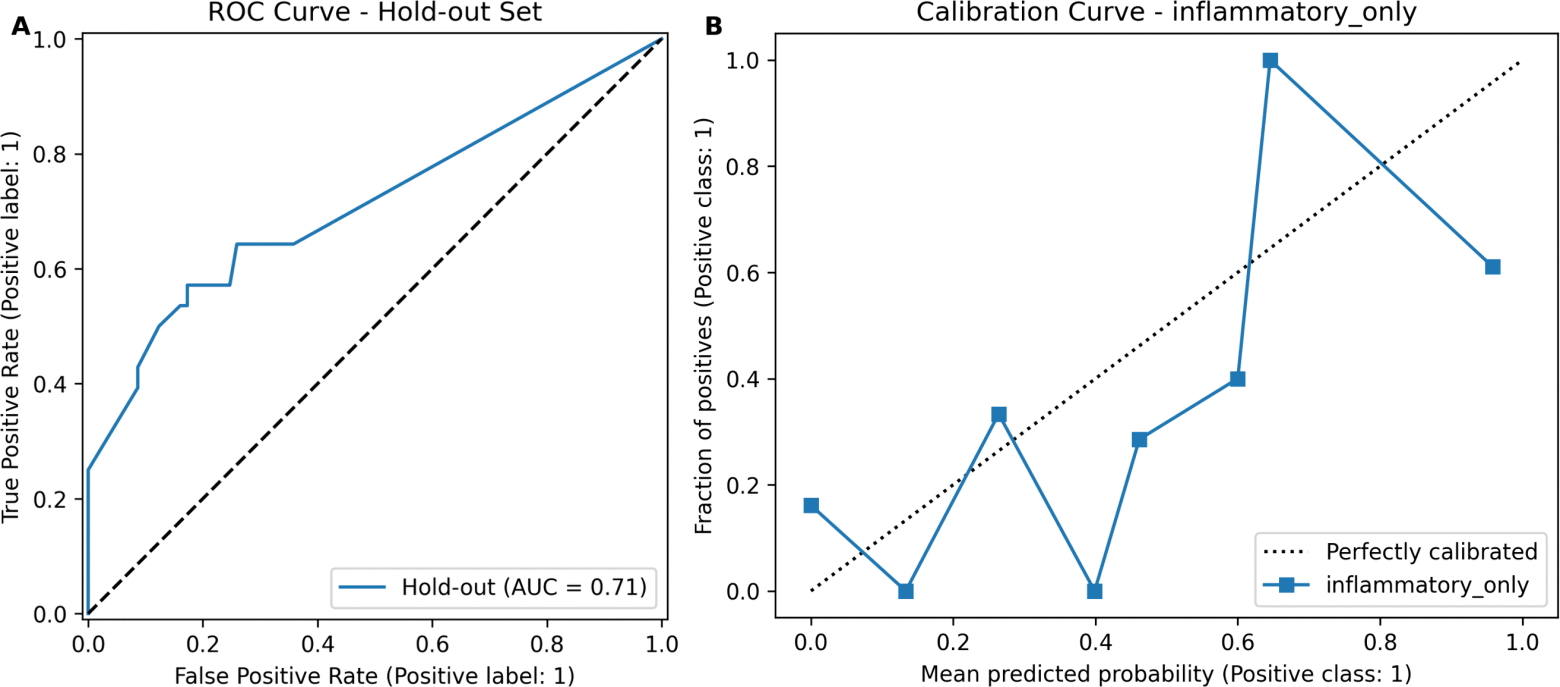
Final validation of the inflammatory panel model in the holdout test set. **(A)** ROC curve (AUC = 0.711). **(B)** Calibration curve demonstrating acceptable agreement between predicted and observed probabilities.

**Fig 2:**
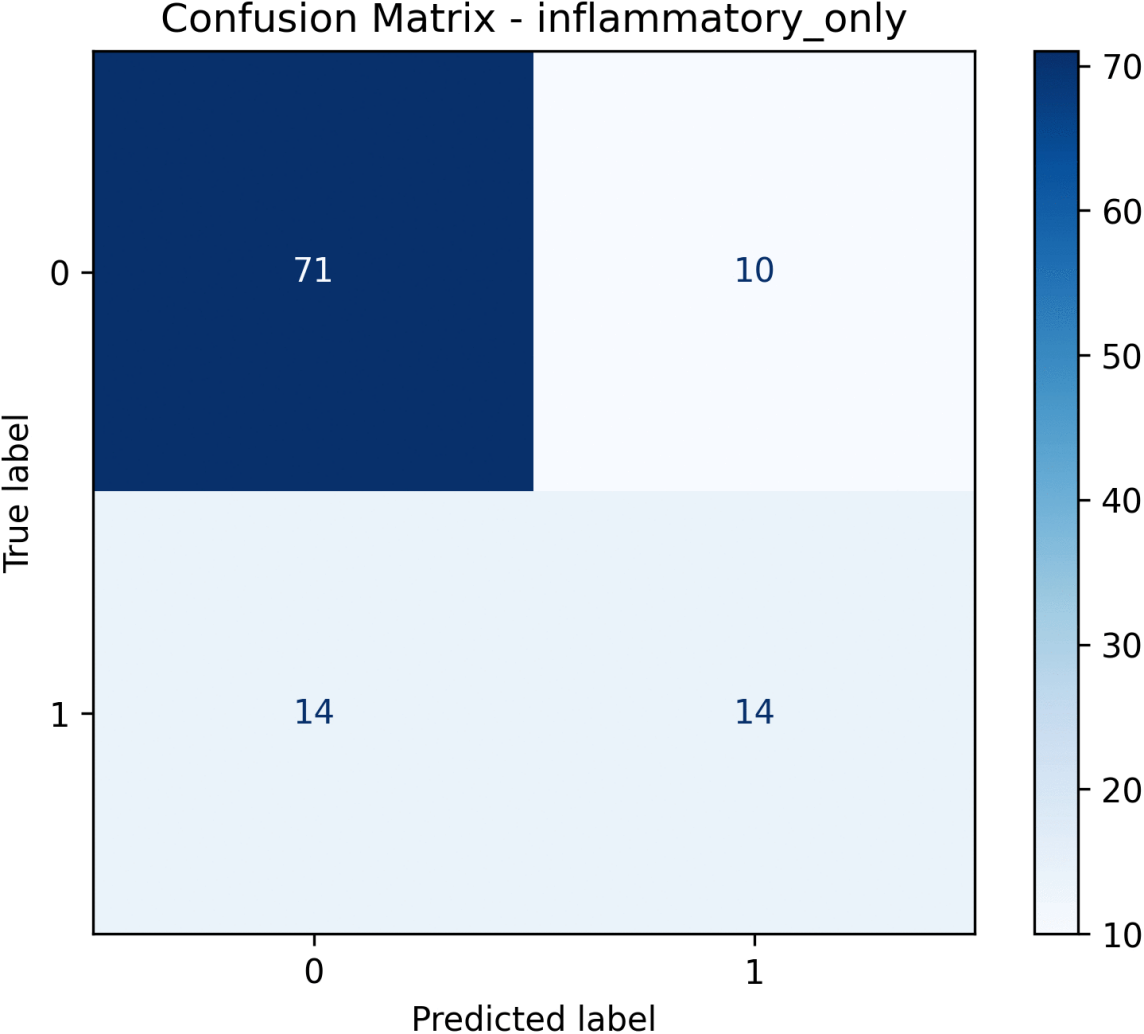
Confusion matrix of the final LightGBM model on the holdout test set.

We then evaluated the final model on an independent holdout test set, where it demonstrated moderate predictive capacity with an AUC of 0.711 (95% CI: 0.591–0.824; Fig 1A). Detailed performance metrics, including precision, recall, specificity, accuracy, and F1 score, together with their corresponding 95% confidence intervals, are summarized in Table 6. The confusion matrix visualizing the model predictions on the holdout set is provided in Fig 2. The calibration curve indicated an acceptable agreement between the predicted probabilities and the actual observations, although minor deviations suggest potential areas for further improvement (Fig 1B). The relatively wide confidence intervals emphasize the importance of future validation in larger datasets to achieve more precise estimates.

**Table 6:** Updated classification metrics of the final model on the holdout test set.

Metric	Value	95% CI
AUC	0.711	0.591–0.824
Accuracy	0.780	0.706–0.853
Specificity	0.877	0.800–0.947
Precision	0.583	0.370–0.792
Recall (Sensitivity)	0.500	0.310–0.682
F1 Score	0.538	0.353–0.692

Metrics were calculated based on the confusion matrix of the holdout test set. Precision represents the proportion of positive identifications that were actually correct. Recall (Sensitivity) measures the proportion of actual positives correctly identified by the model. Specificity measures the proportion of actual negatives correctly identified. The F1 score is the harmonic mean of precision and recall. The Area Under the Curve (AUC) quantifies overall model discrimination

Although the expanded biomarker panel (VIF < 10) included additional relevant features, it yielded slightly lower overall discriminatory power in the holdout set (AUC = 0.699, 95% CI: 0.585–0.809) than the primary inflammatory panel (AUC = 0.711, 95% CI: 0.591–0.824). While the expanded panel demonstrated higher specificity (0.951) and accuracy (0.807), its significantly lower recall (0.393 vs. 0.500) supports the selection of the more parsimonious inflammatory set for early-stage screening, where identifying a greater proportion of at-risk individuals is a priority (S3 Table and S2 Fig).

### 3.3 SHAP interpretation of key biomarkers

**Fig 3:**
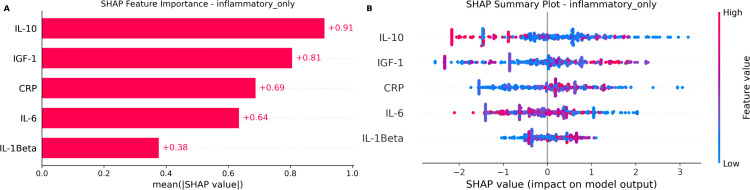
SHAP analysis of the final LightGBM model. **(A)** Global feature importance, ranking predictors by their mean absolute impact. **(B)** Beeswarm plot showing the impact of each predictor’s value on the model output for every individual. The analysis highlights IGF-1, IL-10, and CRP as top influential biomarkers.

SHAP feature importance analysis identified IGF-1, IL-10, and CRP as the most influential biomarkers in the final LightGBM model (Fig 3). These biomarkers consistently demonstrated strong predictive contributions, emphasizing the central role of inflammation in the early metabolic disorder associated with prediabetes. In particular, CRP emerged among the top three biomarkers in both SHAP analyses with multicollinearity thresholds of VIF < 5 and VIF < 10 (see Supplementary S1 Fig), highlighting its consistency as a predictive marker. A comparative sensitivity analysis using a multicollinearity threshold (VIF < 10) (see Supplementary S2 Table) underscored the advantage of our focused inflammatory biomarker approach, as the inclusion of additional correlated biomarkers reduced the interpretability and clarity of the feature importance estimates.

## 4 Discussion

This study successfully identified a panel of inflammatory biomarkers (IGF-1, IL-10, and CRP) that shows potential to predict prediabetes without relying on traditional glycemic markers. Although the holdout validation AUC of 0.711 indicates moderate predictive power, its true significance lies in achieving this performance without any glycemic input. This finding establishes that a distinct inflammatory signal is present and can be independently detected in the prediabetic state, offering a fundamentally new axis for early risk assessment and underscoring the biological importance of our findings.

The identification of inflammatory biomarkers underscores the biological significance of our findings. IGF-1 is recognized for its role in metabolic homeostasis and modulation of inflammation [43]. Previous research highlights the potential to target inflammation in metabolic diseases such as diabetes [44]. IL-10 has been associated with the regulation of chronic inflammation in metabolic disorders [45]. Finally, CRP, a general marker of inflammation, has demonstrated predictive capacity for insulin resistance and the subsequent development of diabetes [46]. The performance of this panel aligns well with the hypothesis that chronic inflammation is a primary driver of insulin resistance that defines prediabetes [47].

A key strength of our methodology was the consideration of multicollinearity among variables to improve the interpretability of our results and minimize the effect of correlation. Using a threshold for VIF < 5, our analysis identified a concise and efficient inflammatory panel. In a sensitivity analysis using a less restrictive threshold of VIF < 10, the panel that included all biomarkers produced a mean cross-validation AUC of 0.727 (95% CI: 0.708–0.747; S2 Table), with GSSG, triglycerides, and CRP emerging as the most critical features (S1 Fig). However, this broader panel did not offer a performance advantage over our more focused inflammatory panel (Mean AUC: 0.727 vs. 0.743; S2 Table and S1 Table, respectively). This highlights a classic trade-off: for this dataset, the simpler inflammatory model provided the best balance of predictive accuracy and interpretability.

Regarding the holdout set, the performance metrics of our model highlight its ideal clinical application. Although the AUC was 0.711 and the F1 score was 0.538 in an imbalanced cohort, the interplay between high specificity (0.877) and moderate sensitivity (0.500) is particularly informative. This profile suggests that the optimal role of the model is not as a standalone diagnostic test but as a highly effective screening tool to identify a subset of at-risk individuals who would most benefit from confirmatory glycemic testing. The successful identification of half of actual prediabetic cases using an independent biological signal represents a significant advance for targeted preventive medicine.

### 4.1 Practical feasibility and implementation considerations

Although identified inflammatory biomarkers (IGF-1, IL-10, and CRP) show promising predictive potential, practical considerations, such as cost, availability, and laboratory requirements, significantly influence their use in routine screening settings. The C-reactive protein (CRP) is the most feasible and practical of the three [48]. However, insulin-like growth factor (IGF-1) testing, although clinically accessible, generally involves higher costs, requires specialized equipment, and presents variability between different assay platforms, thus limiting its widespread implementation [49,50]. The interleukin-10 (IL-10) assay is primarily a research-based test, available only to a limited extent due to its high costs, complex logistics for sample handling, and insufficient standardization, restricting its immediate application in clinical screening [51]. Although these biomarker tests indicate promising predictive accuracy, it should be noted that standard HbA1c tests are generally more cost-effective and widely accessible. Thus, the biomarker panel identified here is expected to complement rather than replace existing diagnostic procedures.

### 4.2 Limitations and future directions

This study has several limitations, each guiding clear directions for future research:

1. **Generalizability and cohort diversity:** A sample of 545 from a single rural location limits the generalizability of our findings. Biomarker expression can vary substantially between diverse ethnic and geographic populations, highlighting the need for external validation in more extensive and diverse study cohorts. Using a biomarker panel with a Variance Inflation Factor of less than 10 could lead to more accurate predictive abilities in larger datasets.
2. **Methodological robustness:** Due to our relatively small sample size, our findings may be affected by the random number seed used for data partitioning. Future research should replicate the analyses using various randomized seed values to confirm the consistency of biomarkers.
3. **Biomarkers interactions and interpretability** Although SHAP highlights the relative importance of individual biomarkers, it does not fully capture the interactions between them. Future research could incorporate advanced interaction methods, such as SHAP interaction values or alternative explainable techniques, to deepen the understanding of how these biomarkers interact biologically and improve predictive models.
4. **Confounding variables and model complexity:** Our study mainly focused on biomarkers, but variables such as age and other demographic characteristics were notable confounders. Future research should create hybrid predictive models that integrate biomarkers with additional demographic and clinical factors, such as BMI and waist circumference. Methods such as stratification by age groups would further clarify the effects of these biomarkers, regardless of confounders.
5. **Biomarker dynamics and advanced modeling:** Our study was cross-sectional, capturing biomarkers at a specific point in time. Future studies should include longitudinal data to better understand their dynamics. Furthermore, exploring advanced techniques such as the synthetic minority sampling technique (SMOTE) [52], Bayesian optimization [53], and adaptive probability thresholds could improve predictive accuracy.
6. **Optimal classification thresholds:** Our analysis used a standard fixed probability threshold of 0.5. Future work should investigate adaptive thresholds, potentially improving metrics such as the F1 score and recall, particularly in datasets with significant class imbalance.

## 5 Conclusion

This study introduces a novel biomarker-based approach for the detection of prediabetes, highlighting inflammatory biomarkers (IGF-1, IL-10, and CRP) as promising early indicators, independent of traditional glucose-based methods. Using interpretable machine learning techniques, our model demonstrated promising predictive performance (AUC = 0.711), establishing inflammation as a biological signal of early metabolic dysfunction. Although further validation in larger, diverse populations and practical considerations such as test standardization and cost must be addressed, our findings offer a meaningful step toward precision diagnostics. Ultimately, incorporating inflammation-focused biomarkers into routine screening protocols could facilitate earlier preventive interventions, significantly reducing the risk of progression to type 2 diabetes.

## Supporting information

S1 File Combined supporting information. This file contains supplementry tables.
(PDF)

S1 FigSHAP analysis of the best model from the VIF<10 analysis: Shows global feature importance (bar plot) and a SHAP summary beeswarm plot identifying GSSG, Triglyceride, and CRP as the top predictors.(TIF)

S2 FigHoldout set validation for the best VIF<10 model: Includes the ROC curve, calibration plot, and confusion matrix for the expanded all-biomarker panel.(TIF)
