# Supplementary material for: An inflammatory biomarker panel for prediabetes classification using interpretable machine learning
Source: PLoS One. 2026 Mar 16;21(3):e0341195. doi: 10.1371/journal.pone.0341195 (PMC12991234; doi:10.1371/journal.pone.0341195)
Supplement: S1 File — This file contains supplementry tables. (PDF) [file pone.0341195.s001.pdf]

Supplementary Materials

The supplementary material provides comprehensive data supporting our main findings and comparative sensitivity analysis.

- **Table S1** details the complete cross-validation results for our primary analysis, which used a multicollinearity threshold (VIF < 5).
- **Table S2** contains the results of the comparative sensitivity analysis using a threshold (VIF < 10).
- **Figure S1** shows the SHAP summary plots for the best-performing model from the VIF < 10 analysis (XGBoost, `all_biomarkers` panel).
- **Table S3 and Figure S2** present the holdout set validation results for the VIF < 10 model, including detailed performance metrics and visualizations.

S1 Table. Full Summary of Cross-Validation Performance (VIF < 5).

Biomarker Panel	Model	Mean AUC	95% CI for AUC	Mean Accuracy	Mean F1 Score
`cholesterol_only`	RandomForest	**0.630**	0.555–0.704	0.670	0.369
	LightGBM	0.586	0.558–0.615	0.592	0.376
	XGBoost	0.587	0.542–0.656	0.697	0.256
	LogisticRegression	0.487	0.460–0.500	0.350	0.366
`oxidative_only`	RandomForest	**0.717**	0.679–0.749	0.720	0.405
	LightGBM	0.690	0.642–0.739	0.665	0.417
	XGBoost	0.699	0.667–0.730	0.739	0.375
	LogisticRegression	0.621	0.563–0.680	0.511	0.423
`inflammatory_only`	RandomForest	0.717	0.658–0.777	0.725	0.436
	LightGBM	**0.743**	0.686–0.802	0.690	0.458
	XGBoost	0.734	0.671–0.797	0.755	0.406
	LogisticRegression	0.555	0.478–0.634	0.569	0.346
`HbA1c_only`	RandomForest	0.579	0.534–0.623	0.596	0.396
	LightGBM	**0.599**	0.543–0.649	0.571	0.390
	XGBoost	0.593	0.535–0.645	0.734	0.000*
	LogisticRegression	0.586	0.505–0.652	0.612	0.453
`all_biomarkers`	RandomForest	0.722	0.679–0.775	0.734	0.425
	LightGBM	**0.734**	0.684–0.792	0.681	0.441
	XGBoost	0.733	0.681–0.787	0.759	0.400
	LogisticRegression	0.660	0.609–0.71	0.560	0.441
`age_only`	RandomForest	0.573	0.545–0.597	0.578	0.387
	LightGBM	0.561	0.528–0.585	0.537	0.390
	XGBoost	0.571	0.557–0.586	0.720	0.133
	LogisticRegression	**0.589**	0.552–0.622	0.539	0.410
`all_features`	RandomForest	0.722	0.679–0.775	0.734	0.425
	LightGBM	**0.734**	0.684–0.792	0.681	0.441
	XGBoost	0.733	0.681–0.787	0.759	0.400
	LogisticRegression	0.660	0.609–0.71	0.560	0.441

AUC: Area under the Receiver Operating Characteristic Curve; CI: Confidence Interval. Metrics calculated using five-fold cross-validation.

*An F1 score of 0.000 indicates the model failed to identify any true positives during cross-validation. This typically occurs when the model's predicted probabilities for the positive class consistently fall below the classification threshold (e.g., 0.5), resulting in zero positive predictions. This outcome is common in highly imbalanced datasets where the model, even when attempting to adjust for imbalance, cannot find a strong enough signal from the feature when it is used in isolation.

S2 Table. Full Summary of Cross-Validation Performance (VIF < 10).

Biomarker Panel	Model	Mean AUC	95% CI for AUC	Mean Accuracy	Mean F1 Score
`cholesterol_only`	RandomForest	**0.639**	0.567–0.710	0.667	0.368
	LightGBM	0.584	0.552–0.615	0.587	0.374
	XGBoost	0.582	0.532–0.652	0.700	0.220
	LogisticRegression	0.487	0.460–0.500	0.350	0.366
`oxidative_only`	RandomForest	**0.720**	0.684–0.756	0.722	0.396
	LightGBM	0.704	0.669–0.739	0.670	0.435
	XGBoost	0.700	0.670–0.731	0.750	0.379
	LogisticRegression	0.634	0.585–0.678	0.535	0.452
`inflammatory_only`	RandomForest	0.721	0.676–0.770	0.725	0.420
	LightGBM	**0.731**	0.683–0.779	0.683	0.431
	XGBoost	0.726	0.662–0.787	0.745	0.364
	LogisticRegression	0.551	0.478–0.630	0.569	0.347
`HbA1c_only`	RandomForest	0.579	0.534–0.623	0.596	0.396
	LightGBM	**0.599**	0.543–0.649	0.571	0.390
	XGBoost	0.593	0.535–0.645	0.734	0.000*
	LogisticRegression	0.586	0.505–0.652	0.612	0.453
`all_biomarkers`	RandomForest	**0.735**	0.721–0.750	0.736	0.415
	LightGBM	0.719	0.699–0.740	0.700	0.453
	XGBoost	0.727	0.708–0.747	0.752	0.414
	LogisticRegression	0.668	0.610–0.726	0.576	0.459
`age_only`	RandomForest	0.573	0.545–0.597	0.578	0.387
	LightGBM	0.561	0.528–0.585	0.537	0.390
	XGBoost	0.570	0.554–0.585	0.720	0.133
	LogisticRegression	**0.589**	0.552–0.622	0.539	0.410
`all_features`	RandomForest	**0.735**	0.721–0.750	0.736	0.415
	LightGBM	0.719	0.699–0.740	0.700	0.453
	XGBoost	0.727	0.708–0.747	0.752	0.414
	LogisticRegression	0.668	0.610–0.726	0.576	0.459

AUC: Area under the Receiver Operating Characteristic Curve; CI: Confidence Interval. Metrics calculated using five-fold cross-validation.

*An F1 score of 0.000 indicates the model failed to identify any true positives during cross-validation. This typically occurs when the model's predicted probabilities for the positive class consistently fall below the classification threshold (e.g., 0.5), resulting in zero positive predictions. This outcome is common in highly imbalanced datasets, where the model, even when attempting to adjust for imbalance, cannot detect a strong enough signal from the feature when used in isolation.

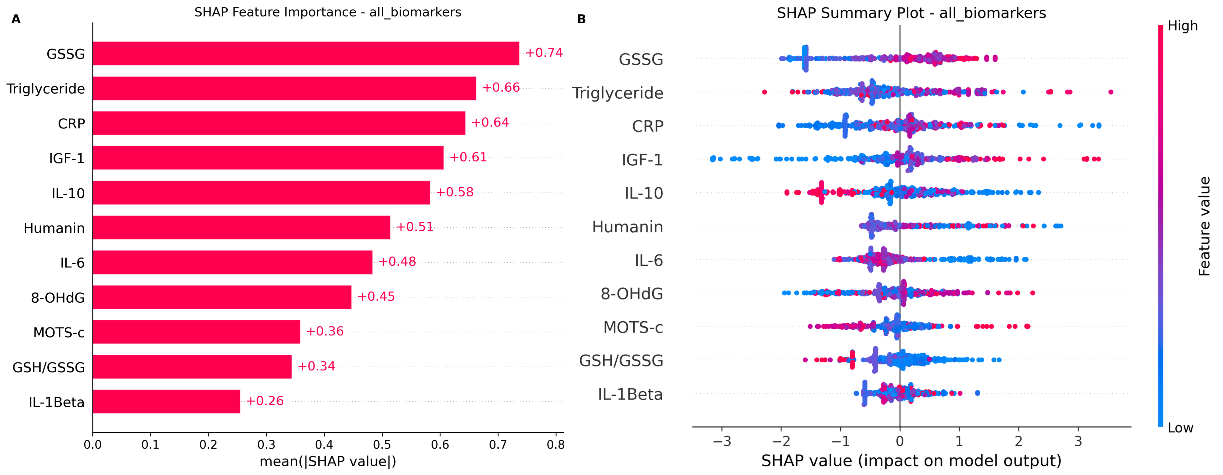

S1 Fig. SHAP analysis of the best model from the VIF < 10 analysis (XGBoost, `all_biomarkers` panel). The combined figure shows global feature importance (bar plot) and a SHAP summary beeswarm plot illustrating the impact of each predictor's value on the model output for every individual. The top predictors include `GSSG`, `Triglyceride`, `p66Shc`, `IGF-1`, and `CRP`.

S3 Table. Performance Metrics of the Expanded Biomarker Panel (VIF < 10) on the Holdout Test Set.

**Metric**	**Value**	**95% Confidence Interval**
Area Under Curve (AUC)	0.699	0.585–0.809
Accuracy	0.807	0.734–0.881
Specificity	0.951	0.901–0.988
Precision	0.733	0.533–0.933
Recall (Sensitivity)	0.393	0.217–0.577
F1 Score	0.512	0.300–0.694

Metrics were calculated based on the holdout test set for the all-biomarker model (VIF < 10). Following S2 Fig Panel C, the model yielded 77 true negatives and 11 true positives out of 109 samples (81 controls, 28 prediabetes). Confidence intervals were estimated using bootstrap resampling with 1,000 iterations.

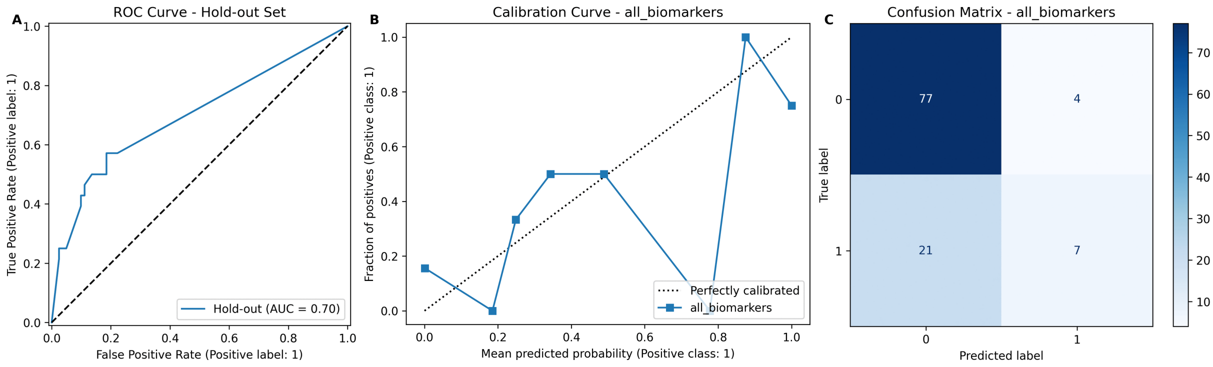

S2 Fig. Holdout set validation for the best VIF < 10 model (XGBoost, `all_biomarkers`). The panels show the ROC curve (AUC = 0.70), the calibration plot comparing predicted and observed probabilities, and the confusion matrix summarizing classification performance on the holdout test set.
