# Supplementary figures and images for: An inflammatory biomarker panel for prediabetes classification using interpretable machine learning
Source: PLoS One. 2026 Mar 16;21(3):e0341195. doi: 10.1371/journal.pone.0341195 (PMC12991234; doi:10.1371/journal.pone.0341195)

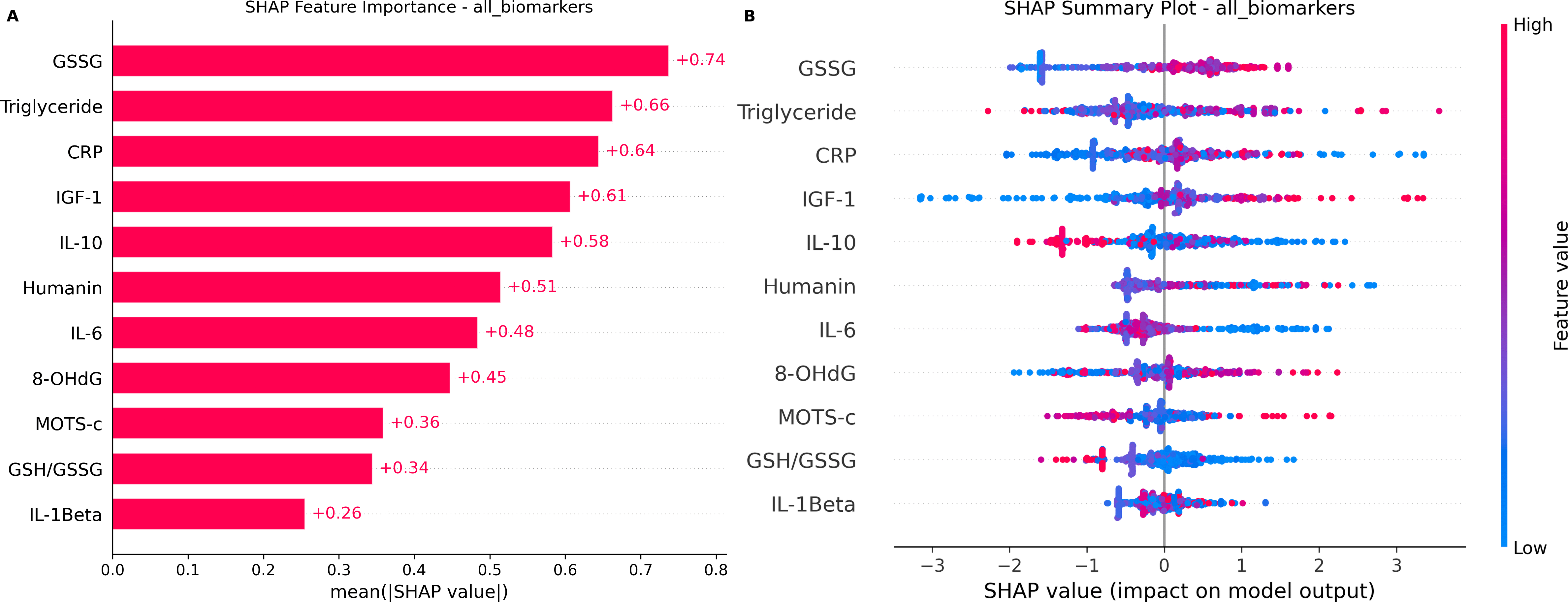

Supplement: S1 Fig — (TIF) [file pone.0341195.s002.tif]

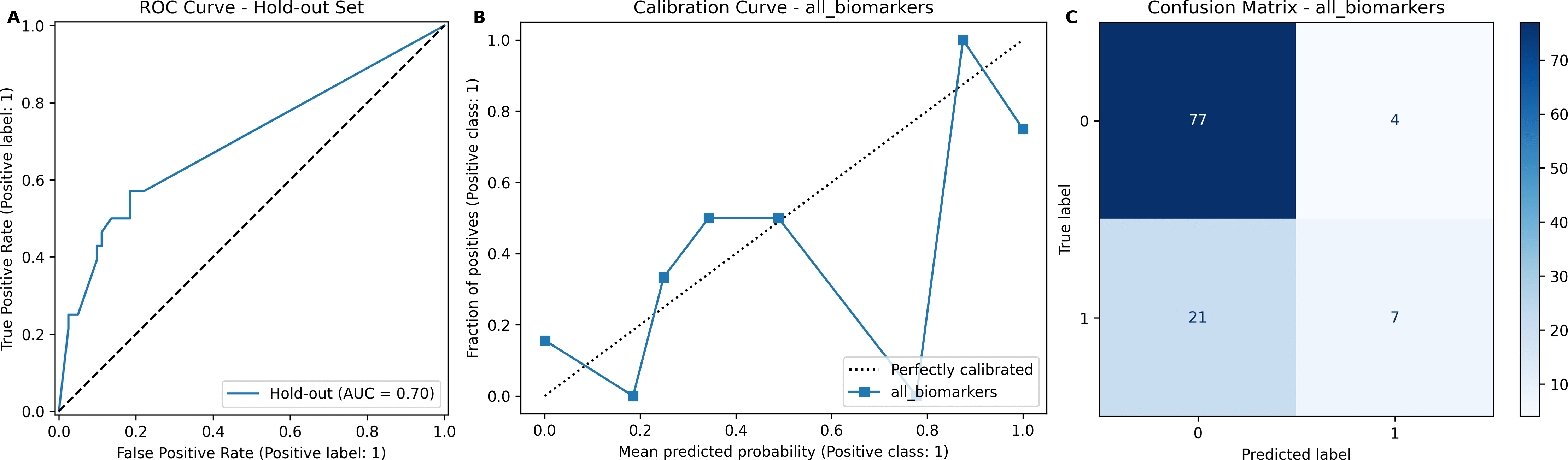

Supplement: S2 Fig — (TIF) [file pone.0341195.s003.tif]
